# Fmoc-Phe : Fmoc-Leu supramolecular hydrogels with adaptive antibacterial activity

**DOI:** 10.1039/d5ra08809g

**Published:** 2026-03-12

**Authors:** Romain Chevigny, Henna Rahkola, Efstratios D. Sitsanidis, Tatu Kumpulainen, Lisa J. White, Lotta-Riina Sundberg, Jennifer R. Hiscock, Mika Pettersson, Maija Nissinen

**Affiliations:** a Department of Chemistry, Nanoscience Center, University of Jyväskylä FI-40014 Jyväskylä Finland maija.nissinen@jyu.fi; b Université de Lorraine, CNRS, IJL F-54000 Nancy France; c School of Natural Sciences, University of Kent Canterbury Kent CT2 7NH UK; d Department of Biological and Environmental Science, Nanoscience Center, University of Jyväskylä FI-40014 Jyväskylä Finland

## Abstract

The development of adaptive soft materials offers new opportunities to address the effects of antimicrobial resistance. Fmoc-phenylalanine (Fmoc-Phe) and Fmoc-leucine (Fmoc-Leu) based hydrogels are known to demonstrate antibacterial activity. We now show that by combining these two gelators (Fmoc-Phe and Fmoc-Leu) into a multicomponent hydrogel system, we can tune the antibacterial properties of the resultant hydrogel. This tunable antimicrobial behaviour is achieved by varying the Fmoc-Phe : Fmoc-Leu ratio, which also influences self-assembly and, as a result, the physical properties of the material. We show that changing the component ratio can be used to optimise gelation efficiency and modulate viscoelastic, self-healing and thermoresponsive properties. Spectroscopic analyses reveal that while β-sheet organisation is retained independently of the ratio of gelators supplied, system stability (increasing material softness) is observed as a direct result of the proportion of the gelator that remains unassembled in the sol of the resultant hydrogel. Antibacterial assays conducted against clinically relevant Gram-positive and Gram-negative pathogens demonstrate formulation-dependent responses, with Gram-positive strains showing the greatest susceptibility. From these data, we can determine a structure–activity relationship, which demonstrates the importance of compositional tuning as a simple and effective strategy for designing peptide-based hydrogels with tailorable physical, material and antimicrobial properties.

## Abstract 2

Dual-component Fmoc-Phe- and Fmoc-Leu-based hydrogels exhibit tunable mechanical properties and thermoresponsiveness. The antibacterial activity against Gram-positive and Gram-negative pathogens can be tuned by varying the building-block ratio.

## Graphical abstract

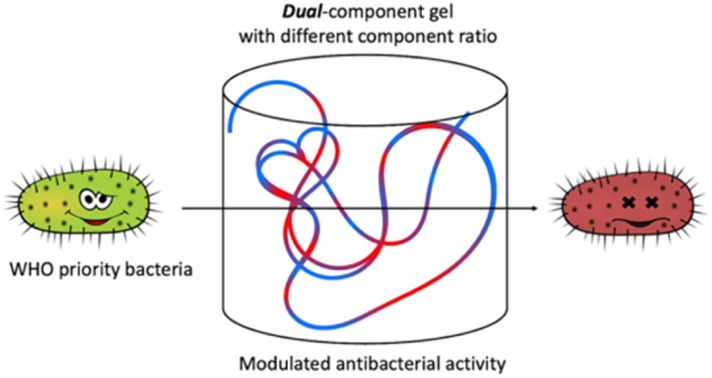

## Introduction

The continued rise of antimicrobial resistance (AMR) is one of the greatest threats to global health.^1–3^ Recent estimates indicate that bacterial AMR is directly associated with nearly five million deaths annually.^4^ Infections caused by Gram-negative pathogens, such as *Klebsiella pneumoniae* (*K. pneumoniae*) and *Escherichia coli* (*E. coli*), and Gram-positive *Staphylococcus aureus* (*S. aureus*), have increased in frequency and severity, leading to high mortality and morbidity.^4^ To address this challenge, new antibacterial materials have been developed, including biomolecules, peptides,^5–7^ nanoparticles,^8–10^ and hydrogels.^11–16^ Hydrogels are particularly attractive because, in addition to any intrinsic antimicrobial properties, they can also be designed to act as local delivery vehicles, concentrating antimicrobials at the infection site while limiting systemic exposure. In addition, hydrogels can serve as adjuvant platforms, enhancing drug efficacy through co-loading with nanoparticles or small-molecule drugs. In addition, peptide-based systems may also provide inherent antimicrobial activity through disruption of bacterial membranes or induction of oxidative stress.^17–21^ Among hydrogel materials, peptide-based supramolecular hydrogels have drawn significant interest due to their inherent biocompatibility, tunable functionality and comparatively low small molecule gelator production costs.^22–25^

Peptide-based supramolecular hydrogels form through the self-assembly of low molecular weight hydrogelators, such as those derived from natural or modified amino acids. These gelators frequently incorporate hydrophobic motifs, such as aromatic residues (*e.g.* phenylalanine, histidine)^26,27^ or aliphatic residues (*e.g.* leucine, alanine)^28,29^ that promote aggregation. This gelator self-assembly is driven by the formation of intermolecular non-covalent interactions combined with solvent–gelator interactions, giving rise to an insoluble fibrillar network that entraps the surrounding solvent (sol) to form a gel. In aqueous systems, hydrophobic interactions and π–π stacking are particularly important, which is why such motifs are often present in the peptide-gelator sequence.^30,31^ To further strengthen these interactions, bulky aromatic protective groups, such as fluorenylmethyloxycarbonyl (Fmoc),^32–35^ naphthalene and pyrene^25,36^ are often incorporated at the N-terminus of peptide hydrogelators, enhancing self-association and promoting hydrogel formation.^37–41^

The use of this class of hydrogels as antibacterial materials has already been demonstrated.^26,42,43^ For example, Fmoc-phenylalanine (Fmoc-Phe) hydrogels have demonstrated antimicrobial activity against both Gram-positive (*e.g. S. aureus*) and Gram-negative bacteria (*e.g. E. coli*).^13,24,27^ The enhanced activity of these hydrogels against Gram-positive over Gram-negative bacteria is attributed to the lipopolysaccharide-containing outer membrane present in Gram-negative bacteria.

The use of multicomponent hydrogels (defined as hydrogels containing more than one gelator) is growing in popularity, as this simple approach yields novel materials with properties, such as enhanced antimicrobial efficacy, that would not be achieved by single-component systems.^29,37,44,45^ For instance, Fmoc-leucine (Fmoc-Leu) also exhibits antimicrobial activity.^46–48^ Therefore, it is hypothesised that combining Fmoc-Leu with Fmoc-Phe will yield either additive or synergistic enhancement of antimicrobial activity compared to a homogenous hydrogel.^49^ While most studies on multicomponent gelator systems focus on varying the chemical nature of the components to tailor properties, simple changes in the relative ratio of components can also impart distinct and emergent behaviours, an approach that remains comparatively underexplored.^50,51^

Building on this rationale, we investigate dual-component hydrogels composed of Fmoc-Phe, a well-studied antibacterial hydrogelator, and Fmoc-Leu, which has been associated with antimicrobial activity in peptide assemblies. Our aim was to determine how a simple variation of the Fmoc-Phe : Fmoc-Leu molar ratio influences supramolecular assembly, material properties, and antibacterial activity. To this end, we employed infrared and fluorescence spectroscopy to probe molecular-level organisation, and rheology to characterise viscoelastic behaviour. Antibacterial activity was evaluated against a six-strain panel comprising *E. coli*, *K. pneumoniae*, and *P. aeruginosa* (Gram-negative) and *S. aureus*, *B. subtilis*, and *E. faecalis* (Gram-positive; strain available in the experimental section below). This panel includes three WHO-priority/ESKAPE pathogens (*K. pneumoniae*, *P. aeruginosa*, *S. aureus*),^52^*E. coli*, and two Gram-positive comparators (*E. faecalis*, *B. subtilis*). We validated our hypothesis that varying the Fmoc-Phe : Fmoc-Leu ratio modulates supramolecular packing and fibrillar network formation, thereby tuning viscoelasticity, self-healing, and thermoresponsiveness, which in turn govern antibacterial efficacy. This provides insight into the use of a relatively simple approach to develop novel weapons in the fight against AMR infections.

## Results and discussion

### Gel preparation

**Fig. 1:**
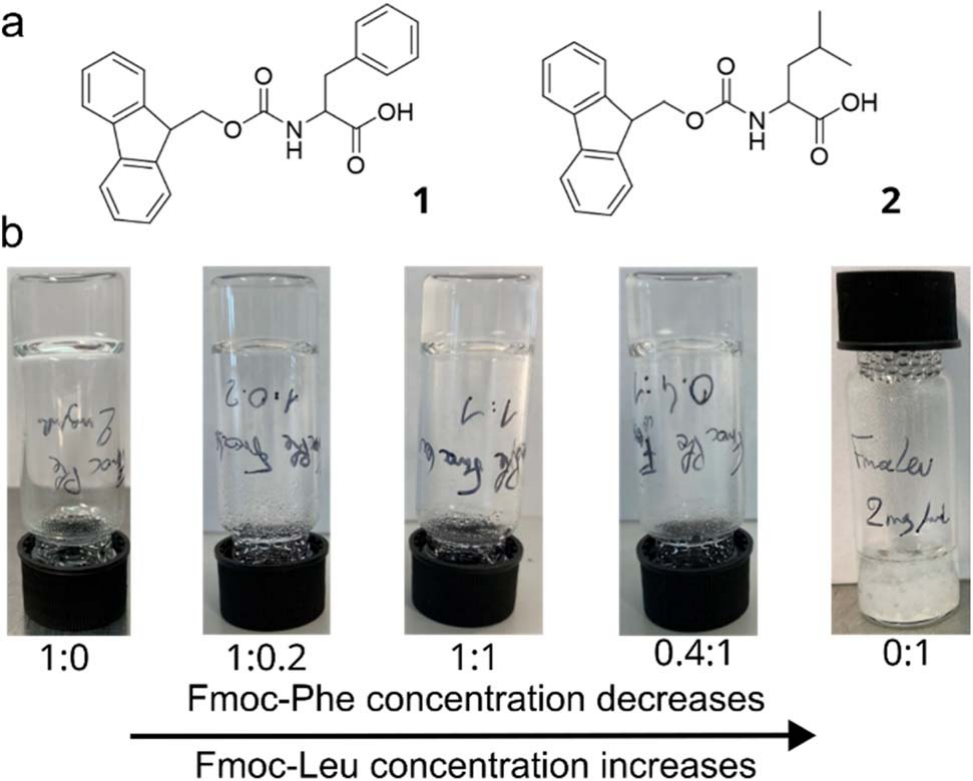
(a) Chemical structures of Fmoc-Phe (1, left) and Fmoc-Leu (2, right). (b) Vial inversion screening of dual-component hydrogels at varying 1 : 2 ratios. The concentration of Fmoc-Phe decreases, and that of Fmoc-Leu increases from left to right. The 0 : 1 ratio yields a suspension.

Fmoc-Phe (1) forms a translucent supramolecular gel in phosphate buffer saline (PBS) solution *via* a heating/cooling pathway (the gelation protocol is presented in the Experimental section below). The dual hydrogels were formed by varying the ratio of 1 and 2 (Fmoc-Leu), while keeping the total gelator concentration constant (Table 1, Fig. 1). The minimum gelation concentration (MGC) was determined by inversion test and confirmed by rheological studies (Fig. S9), and it was found to be 2 mg mL^−1^ of total gelator concentration. All ratios, except Fmoc-Leu alone (ratio 0 : 1), yielded self-supporting hydrogels (SSG). The time required to obtain a dual-component SSG increased from 10 min to a few hours when varying the 1 : 2 ratio from 1 : 0 to 0.4 : 1. Increasing the Fmoc-Leu concentration in the system increased the gelation time of the dual-component material.

**Table 1:** Initial gelation concentration screening of dual hydrogels with varying component ratios, corresponding masses and gelation outcomes (vial inversion method confirming lack of free gravitational flow. SSG formation is verified by rheology)

Fmoc-Phe : Fmoc-Leu ratio	Fmoc-Phe : Fmoc-Leu (mg)	Gelation outcome
1 : 0	2 : 0	SSG, transparent
1 : 0.2	1.66 : 0.33	SSG, transparent
1 : 1	1 : 1	SSG, transparent
0.4 : 1	0.57 : 1.42	SSG, transparent
0 : 1	0 : 2	Precipitate

### Mechanical, morphological, self-healing and thermal properties

**Fig. 2:**
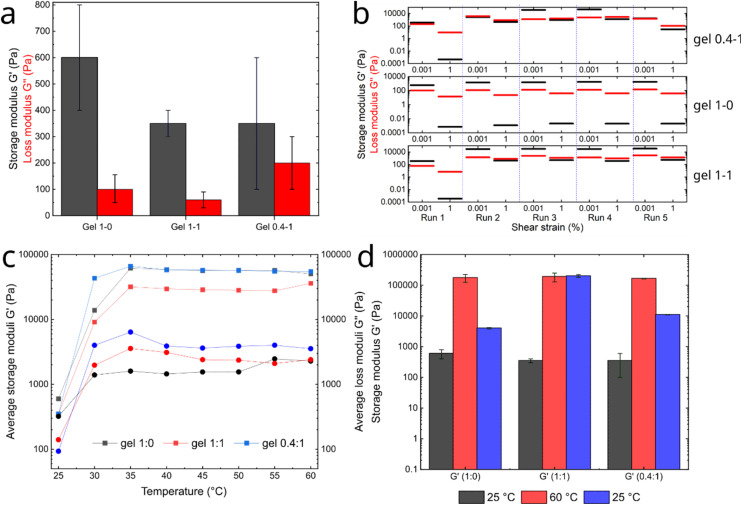
Rheological characterisation of hydrogels prepared at 1 : 0, 1 : 1 and 0.4 : 1 ratios of 1 : 2. (a) Bar graph produced from frequency sweeps (*n* = 3) data showing *G*′ (black) and *G*″ (red) values. (b) Continuous step strain measurements obtained from frequency sweeps measurements over five runs (*n* = 3) of repetitive gel breaking/reformation cycles. Each hydrogel sample was allowed to rest for 15 min between runs to allow reformation. Values shown were extracted at the plateau for the lowest and highest shear strain values obtained. (c) *G*′ and *G*″ data obtained from temperature-controlled frequency sweeps (*n* = 3) ranging from 25 °C to 60 °C. (d) Data obtained from frequency sweep measurements (*n* = 3) at 25 °C (black-initial temperature), 60 °C (red-heating) and 25 °C (blue-cooling) demonstrating the thermal reversibility of these materials.

Rheological experiments were performed on the dual-component system at different 1 : 2 ratios (1 : 0, 1 : 1, and 0.4 : 1) to confirm gelation and to determine the effect of varying gelator ratios on the mechanical properties of the resultant hydrogels. Amplitude sweep (AS) measurements were first recorded on freshly prepared hydrogels to verify the viscoelastic behaviour of the materials and determine the linear viscoelastic region (LVR). To ensure reproducibility, all measurements were performed in triplicate. The storage modulus *G*′ (or elastic modulus, solid-like nature) was found to be higher than the loss modulus *G*″ (or viscous modulus, liquid-like nature) for all treated samples, confirming the viscoelastic nature of the hydrogels^35,53^ (Fig. S9). From the AS measurements, a shear strain (*γ*%) of 0.00464% within the LVR was used to perform frequency sweep (FS) measurements. Hydrogels at the 1 : 0 ratio showed a *G*′ of ∼600 Pa, while those at 1 : 1 and 0.4 : 1 ratios appeared softer with a *G*′ of ∼350 Pa (Fig. 2a), resembling the stiffness of the brain tissue.^54,55^

As the amount of Fmoc-Phe decreases, the material's stiffness decreases, indicating that the strength of the network is compromised. To visually support the change in behaviour with the ratio, transmission electron microscopy (TEM) images of the gels were recorded (Fig. S13). The fibre network of gel at 1 : 0 ratio is composed of thick and straight needle-like fibres, entangling in bigger bundles. Upon increasing the content of 2, an evolution to thinner, more curved and entangled fibres is observed (ratio 1 : 1), which ultimately leads to the formation of platted ribbons and flat fibres together with thin fibres (ratio 0.4 : 1). These observations suggest a change in the higher-order organisation of the gelators, more specifically to the third-order organisation (see chapter Molecular and higher-order organisation for discussion on the secondary organisation), which correlates with a significant difference in rheological properties and stiffness.

Continuous step strain measurements were subsequently performed over five runs of breaking and reformation cycles to correlate material stiffness with potential self-healing properties (the ability of the gel to naturally recover its supramolecular network after rupture; Fig. 2b). The Fmoc-Phe hydrogel (1 : 0) demonstrated excellent reproducibility and stability of its storage modulus over the five runs (return to the initial *G*′ value every run), indicating a good self-healing ability. For the 1 : 1 dual-component hydrogel, an increase in the stiffness of approximately 30 kPa was observed during the second run. This increase remained constant until the fifth run, suggesting that the reformed network (network broken after the first run at a high shear strain) was stronger than the original before any mechanical stress was applied. A similar trend was observed for the lowest ratio, 0.4 : 1, of the dual-component system. Indeed, the *G*′ increased during the third run and decreased at the last. The value proximity of the *G*′ and *G*″ moduli and the lack of reproducibility during the runs for this material also denotes a weaker network, possibly due to the comparative decrease in π–π interactions.

The general trend suggests that these materials exhibit higher stiffness under mechanical stress. Additionally, the ability to self-heal is significantly reduced as the proportion of 2 increases. The thermal behaviour of the materials was also assessed by temperature-controlled frequency sweeps. These data were recorded at temperatures ranging from 25 °C to 60 °C at 5 °C intervals. The hydrogels were allowed to rest from mechanical stress for ∼15 min at the measurement temperature between each run. The changes in *G*′ as a function of temperature are shown in Fig. 2c (full data set is available in Fig. S10–S12). For all gel systems, an increase in *G*′ (20–30 fold) was observed, starting at 30 °C and plateauing between 35 °C and 60 °C. The *G*″ also increased and plateaued similarly, with a value below that of *G*′, verifying the viscoelastic behaviour at high temperature. This trend is similar to that observed for the same materials under mechanical stress (continuous step strain measurements, Fig. 2b), demonstrating the stimuli-response properties of the hydrogels to both mechanical and thermal stimuli.

To verify the reversibility of this behaviour, frequency sweep measurements were carried out on the same hydrogels after cooling to 25 °C (Fig. 2d). The thermal response of the materials (1 : 0 and 0.4 : 1) was qualitatively reversible after reaching the initial temperature; however, this was not observed for the 1 : 1 gel. This indicates that the gelators were initially encapsulated in the solvent (before heating), possibly self-assembled after heating and cooling down to the initial temperature.

### Molecular and higher-order organisation

The proportions of 1 and 2 in solution and in the self-assembled solid fibrous state were quantified using variable-temperature ^1^H NMR spectroscopy (VT-NMR). Measurements were performed over the temperature range of 30 °C to 70 °C in 5 °C increments, with a 5 minute equilibration time between steps.

**Fig. 3:**
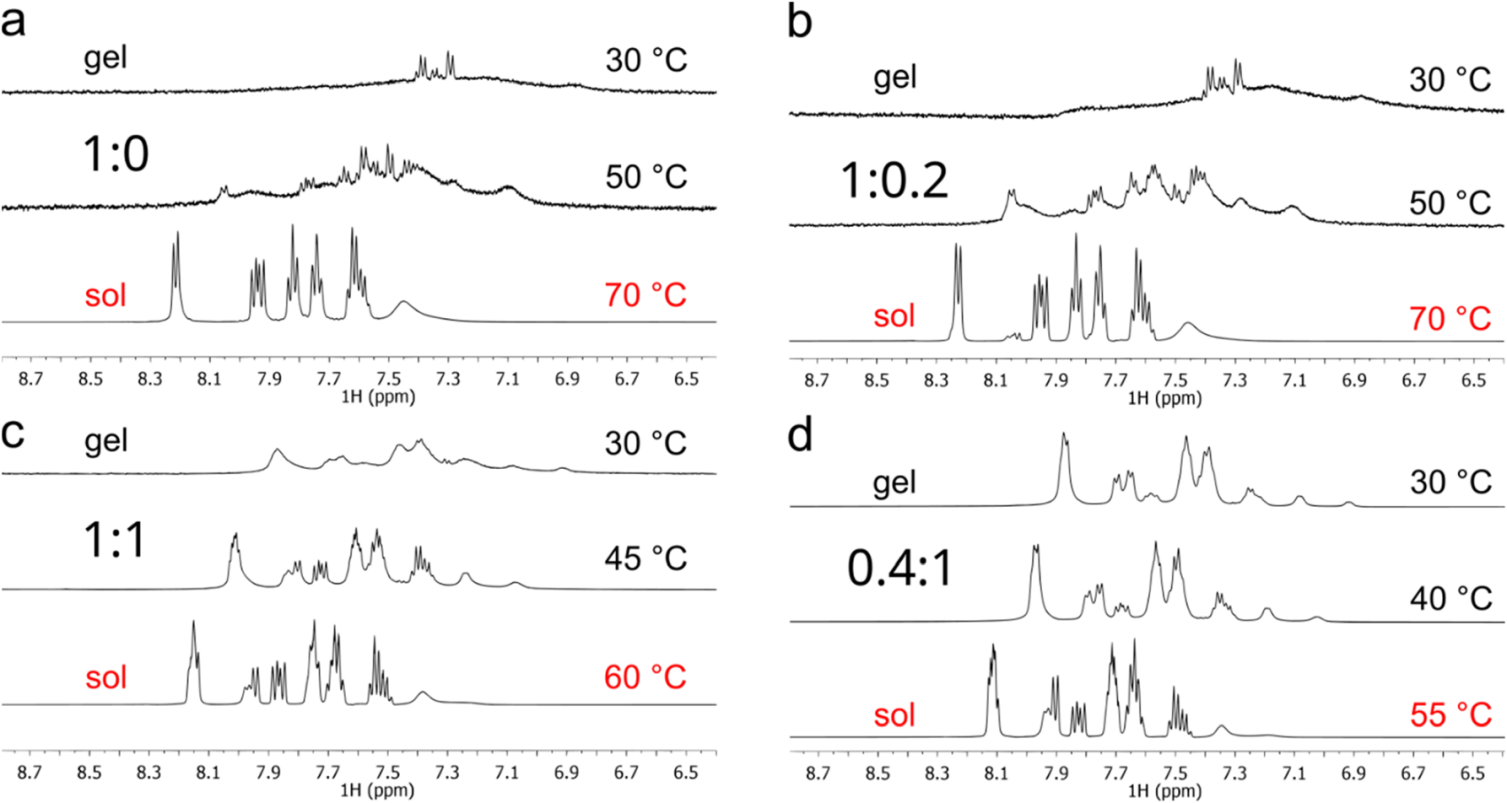
Variable temperature NMR spectra of Fmoc-Phe : Fmoc-Leu gel system at (a) 1 : 0, (b) 1 : 0.2, (c) 1 : 1 and (d) 0.4 : 1 ratios. Hydrogels formed *in situ* in D_2_O-PBS in the NMR tubes and were measured on the day of gelation. Spectra were recorded after a 5 min equilibration time between measurements.

Four different gel ratios were explored (Fig. 3; full dataset provided in Fig. S3). NMR spectroscopy allows for the detection of freely rotating compounds in solution. Upon self-assembly and resulting size increase, the free rotation of the aggregate is reduced, causing the NMR signals to broaden. If the tumbling rate of the entities involved in self-assembly exceeds the detection limit of solution-state NMR spectroscopy, these molecules become NMR-silent (undetectable). At 30 °C, for the hydrogel containing only gelator 1 (Fig. 3a), the presence of low-intensity signals at the baseline, rather than intense multiple peaks, indicates that most gelator molecules have a reduced ability to rotate in the medium. Upon increasing the temperature to 50 °C and then 70 °C, the proton signals increase in intensity and become distinct. This indicates that the gelators are no longer held in an aggregated state, but are freely diffusing in the sol, causing the hydrogel to degrade.

In the dual-component hydrogel systems, the addition of Fmoc-Leu led to more distinct NMR signals at 30 °C, with increasing distinction as the proportion of Fmoc-Leu increased (1 : 0.2 (Fig. 3b), 1 : 1 (Fig. 3c), 0.4 : 1 (Fig. 3d)). Additionally, the increase in signal intensity and the decrease in broadness were observed at higher temperatures, indicating reduced self-assembly and material degradation. The observed spectral changes correlate with the trend in the phase transition temperature (*T*_gel–sol_, Table S1), which decreased from 60 °C for the Fmoc-Phe standalone gel (1 : 0 ratio) to 45 °C upon addition of Fmoc-Leu (0.4 : 1 ratio). While VT-NMR spectroscopy does not directly probe the macroscopic gel–sol transition, these results suggest a temperature-dependent disassembly process which is consistent with the rheological behaviour observed for these thermally responsive hydrogels.

It is also important to note that the spectral profiles of the gel systems at the initial temperature (30 °C) differ, despite all materials being self-supporting hydrogels. As the Fmoc-Leu concentration increases, the proton resonances become markedly sharper, indicating greater molecular mobility within the system. This suggests that a larger fraction of the gelator molecules remains in a relatively dynamic or solvated environment at the time of gel formation. Consequently, fewer molecules are likely incorporated into higher-order self-assembled structures, resulting in a weaker overall network, as supported by the rheological data. We hypothesize that although Fmoc-Leu contributes to the self-assembly process to some extent, a subset of molecules remains mobile, which may contribute to materials with lower thermal resistance and poorer mechanical properties (see Mechanical behaviour section).

**Fig. 4:**
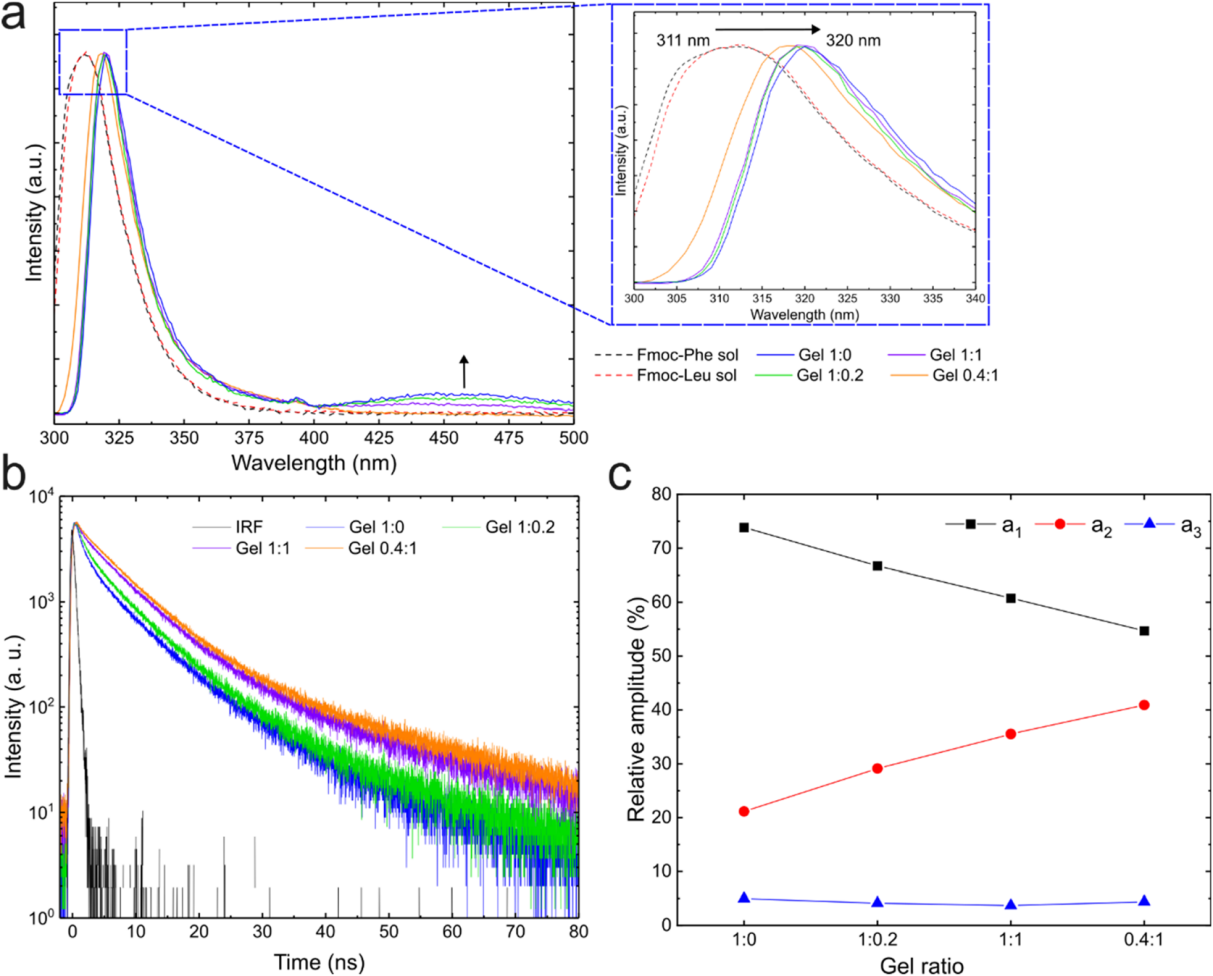
The results obtained from fluorescence spectroscopy studies. (a) Steady-state spectra obtained on compounds in solution and hydrogels in the range 300 nm to 500 nm. Inset is a magnified region between 300 nm and 340 nm. (b) Time-correlated single photon counting (TCSPC) measurements of the four hydrogels. The excitation wavelength was 289 nm, and the hydrogels were formed *in situ* in the quartz cuvettes (1 cm path length) at room temperature. (c) Results of the three-exponential fit of the anisotropy decay curves in (b). Amplitude a1 corresponds to the sub-population in self-assembly or gel state, a2 to the sub-population free in solution and a3 to the sub-population of excimers.

Next, fluorescence spectroscopy was employed to study the interactions between the fluorescent Fmoc protective groups of the gelators. Steady-state fluorescence spectra of the two compounds at all four gel ratios were recorded over 300–500 nm, with an excitation wavelength of 289 nm, as determined from the absorption spectra (Fig. S4). The fluorescence emission maximum was determined at 311 nm for 1 and 2 in solution in their monomeric forms. Upon gelation, a redshift to 320 nm was observed for the gel samples at ratios 1 : 0, 1 : 0.2 and 1 : 1, and to 318 nm for the sample at a 0.4 : 1 ratio (Fig. 4a). The fluorescence maxima correspond to the emission of the π–π* transition of the Fmoc groups.^43,56^ The observed redshift is induced by the overlap of the π orbitals (π–π stacking) during self-assembly, as reported previously.^57,58^ This shift indicates the formation of J-type aggregates, which is also well documented for Fmoc-containing gelators.^59,60^ The difference in redshift observed for the gel sample at a 0.4 : 1 ratio, which we hypothesise is due to the high amount of non-self-assembled species in the solvated phase, implying less extensive π–π stacking. Another interesting feature in the gel spectra appeared at approximately 455 nm, which is attributed to excimer formation.^58,61^ As this band is not shown in the spectra of the monomers, it is associated with the self-assembly process. Interestingly, the excimer emission band decreases as the Fmoc-Phe amount decreases.

Time-correlated single-photon counting (TCSPC) spectroscopy was employed to investigate the fluorescence properties in greater detail. The dilute solution of the monomeric compounds was measured in PBS solution in a quartz cuvette upon excitation with a 290 nm pulsed LED laser. The fluorescence spectra were collected at a right-angle geometry and detected at the magic angle polarisation. The instrument response function (IRF), reflecting the time resolution of the instrument, was measured at parallel polarisation from the scattered excitation light in deionised water. In this case, the IRF is approximately 800 ps under 290 nm UV-LED excitation. For the monomers, the fluorescence decay curves were analysed using a two-exponential function (Fig. S5 and Table S3), revealing the presence of two decay components. The major decay component (approximately 75% amplitude regardless of the monomer) has a fluorescence lifetime of around 6.5 ns, while the minor decay component (approximately 25% amplitude) has a fluorescence lifetime of 500–600 ps. The minor decay component is significantly faster than the time resolution of the instrument and is most likely due to a relaxation process (vibrational or solvent relaxation). The equivalent fluorescence lifetime observed for the major decay component is expected since the fluorophore is the same (Fmoc group in both monomers). The amino acid side chains are non-fluorescent.

Fig. 4b shows the fluorescence decay curves of the hydrogels at different ratios. In this case, a three-exponential function was required to adequately model the data (Table S4). Two of the three decay components show lifetimes similar to those of the monomers but are accompanied by an additional longer-lived component (>12 ns). The decay component with a monomer-like fluorescence lifetime (∼6 ns) corresponds to free molecules in the solvated phase within the gel matrix, and its amplitude increases by up to 40% as the amount of 1 present decreases (Fig. 4c). In contrast to the monomers, the IRF-limited (<800 ps) component has the largest amplitude, which decreases as the amount of 1 decreases. In addition to relaxation processes, the fast-decay component reflects the fraction of molecules involved in self-assembly, whose fluorescence is quenched by intermolecular π–π interactions.^62^ The third sub-population with a >12 ns lifetime is present only in the gel samples and is attributed to the presence of excimers, which can also be observed in the steady-state fluorescence spectra at about 460 nm. Although the amplitude of this decay component remains relatively constant (about 5%), the intensity of the long-wavelength fluorescence band is significantly reduced upon decreasing the amount of Fmoc-Phe (Fig. 3a). Therefore, fluorescence lifetime analysis may quantify subpopulations of the multicomponent system and provide insights into intermolecular interactions within it. Although the major subpopulation within the self-assembly exhibits a lifetime within the IRF, the evolution of the relative amplitudes shows a clear trend.

**Fig. 5:**
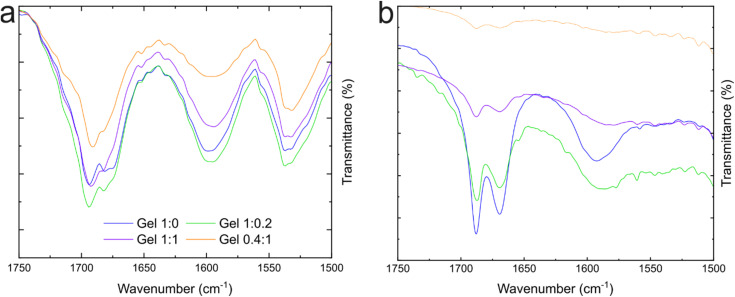
FTIR spectra measured in (a) ATR and (b) transmission modes. Spectra were acquired on freshly prepared samples the day of measurement.

Fourier transform infrared (FTIR) spectroscopy was then employed to characterize the secondary arrangement of the gelators and to provide insights into the intermolecular interactions driving self-assembly. Gels at ratios 1 : 0, 1 : 0.2, 1 : 1 and 0.4 : 1 were freshly prepared and dried at room temperature prior to spectral acquisition in attenuated total reflectance (ATR) mode (Fig. 5a). The resulting spectra were qualitatively similar irrespective of the 1 : 2 ratio. This suggests that whether Fmoc-Leu co-assembles or not, it does not affect the overall structure or the higher-order organisation of the fibres. Given the pronounced propensity of the Fmoc moiety for π–π stacking, the supramolecular architecture is expected to remain largely unchanged upon incorporation of an additional Fmoc-protected amino acid. The amide I region of the spectrum (1600–1700 cm^−1^) contains information on the secondary organisation of the gelators within the fibres.^63,64^ For all gels, the amide I band can be deconvolved into three distinct vibrations (Fig. S8a–d) at 1694 cm^−1^, 1681 cm^−1^ (β-sheet arrangement),^65^ and 1672 cm^−1^ (β-turn). The β-sheet arrangement is expected for supramolecular systems comprising Fmoc protecting groups and phenylalanine units.^25^ Calculating the relative percentage of the band areas allows us to estimate the proportion of each arrangement within the fibres (summarised in Fig. S8e). These proportions were approximately 55% and 15% for the β-sheet and 30% for the β-turn, respectively, for the Fmoc-Phe standalone gel (1 : 0 ratio). Upon decreasing the amount of Fmoc-Phe, the relative area of the 1694 cm^−1^ band increases while that of the 1681 cm^−1^ and 1672 cm^−1^ bands decreases (Fig. S8f). The results suggest that the presence of Fmoc-Leu in the system destabilises the β-turn arrangement in favour of the β-sheet.

As ATR-FTIR spectroscopy is performed on xerogel samples, structural rearrangement can occur. To overcome this limitation, FTIR spectroscopy was also carried out in transmission mode. For this, hydrogels were prepared by two routes: preformed hydrogels were pipetted between two CaF_2_ windows and let to rest for 15 min for the hydrogels to reform, or the hot pre-gel solution was pipetted between the windows, and the hydrogels were formed *in situ*. The spectra of both samples were recorded under identical measurement conditions (see the Experimental section). Both methods resulted in similar spectra (Fig. 5b and S7). Therefore, the higher-order organisation of fibres remains the same regardless of the preparation method, which correlates with the rheology data from breaking-and-reforming cycles (Fig. 2b). Two major bands are observed within the amide I region at 1688 cm^−1^ and 1670 cm^−1^, corresponding to β-sheet and β-turn secondary structures, respectively. FTIR spectroscopy, in both modes, demonstrated that, regardless of the gel ratio (*i.e.*, the proportion of different amino acid side chains), the gelators adopted similar conformations.

### Antibacterial activity

**Fig. 6:**
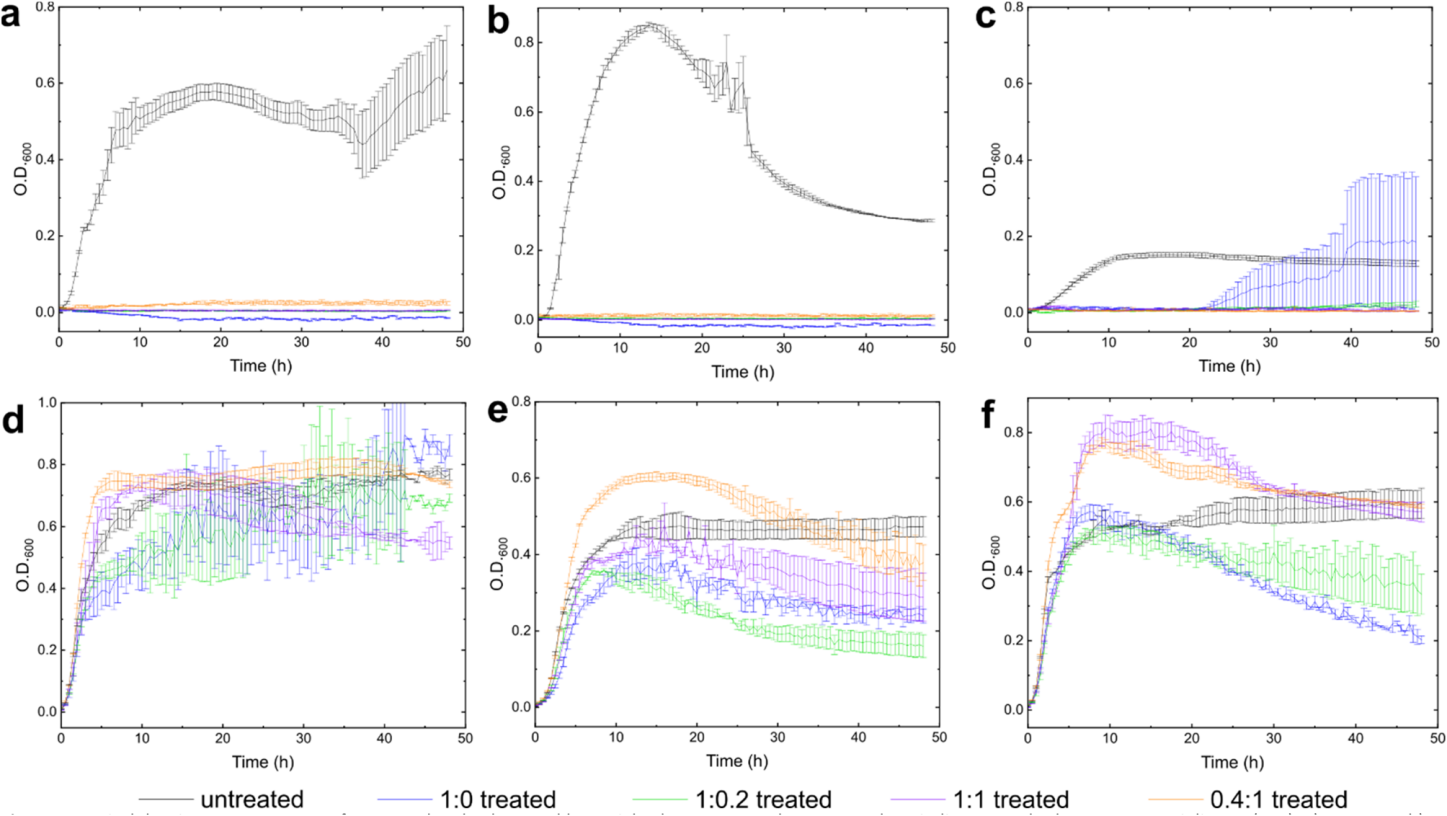
Optical density measurements of untreated and gel-treated bacterial cultures over 48 hours. Error bars indicate standard error across triplicates (*n* = 3). (a) *S. aureus*, (b) *B. subtilis*, (c) *E. faecalis*, (d) *E. coli*, (e) *P. aeruginosa* and (f) *K. pneumoniae*. Hydrogel concentration was 0.2 wt% total gelator.

The antimicrobial potential of the dual-component hydrogels against both Gram-positive and Gram-negative bacteria was evaluated. The four gels, with varying amino acid ratios, were used to investigate the effect of different amino acid compositions on bacterial inhibition. The test panel included *E. coli*, *P. aeruginosa*, *K. pneumoniae*, *S. aureus*, *B. subtilis*, and *E. faecalis*, representing both bacterial classifications (stains in Experimental section below). The antimicrobial efficacy was assessed by monitoring bacterial growth *in vitro* over 48 hours. Optical density at 600 nm (OD_600_) was recorded continuously to observe growth trends in both untreated and hydrogel-treated bacterial cultures (Fig. 6). This approach provides insights into the comparative antimicrobial efficacy of each gel formulation.

The hydrogel formulations exhibited significant antimicrobial activity against the tested Gram-positive strains (using 0.2 wt% hydrogel concentration). For *S. aureus* and *B. subtilis*, all four gel formulations effectively suppressed bacterial growth throughout the 48 hour incubation period, as indicated by near-zero OD_600_ values in treated cultures. This robust inhibitory effect suggests a high susceptibility of Gram-positive bacteria to the dual-component Fmoc-Phe : Fmoc-Leu hydrogels. In the case of *E. faecalis*, initial growth suppression was observed in all formulations, with hydrogels at 1 : 0.2, 1 : 1, and 0.4 : 1 ratios, maintaining lower OD_600_ values throughout the experiment. The Fmoc-Phe standalone gel (1 : 0), however, displayed limited long-term effectiveness, with a modest increase in OD_600_ after 30 hours. These results imply that, while each hydrogel formulation is broadly effective against Gram-positive bacteria, optimising the Fmoc-Phe : Fmoc-Leu ratio may enhance efficacy against specific strains such as *E. faecalis*.

The efficacy of the hydrogels against Gram-negative bacteria was more variable. For *E. coli*, the 1 : 1 gel exhibited the highest degree of growth suppression, though it did not fully prevent bacterial proliferation. Gel 0.4 : 1 demonstrated the least inhibition effect, with OD_600_ values close to those of untreated controls, indicating limited efficacy for this formulation. The growth of *P. aeruginosa* was partially inhibited, with hydrogels at ratios 1 : 0 and 1 : 0.2 providing the strongest suppression, while hydrogels at ratios 1 : 1 and 0.4 : 1 allowed substantial bacterial growth, thus suggesting a reduced antimicrobial effect. For *K. pneumoniae*, the Fmoc-Phe standalone hydrogel (1 : 0) was notably more effective, maintaining lower OD_600_ values than other hydrogels. Gel 0.4 : 1 provided minimal inhibition, with OD_600_ values comparable to those of untreated samples. These observations suggest that although some hydrogel formulations have moderate activity against Gram-negative bacteria, overall efficacy may be enhanced by adjusting the Fmoc-Phe : Fmoc-Leu ratio to optimise bactericidal effects.

The initial growth rates over the first 10 hours reveal interesting trends across different bacterial strains and treatments. For Gram-positive bacteria *S. aureus* and *B. subtilis*, all treated samples showed minimal or no initial growth, indicating that each formulation rapidly inhibited bacterial proliferation from the start of the experiment. This suggests a potent, immediate antimicrobial effect of the hydrogels against Gram-positive strains that persists for at least 48 hours. Gram-negative bacteria *E. coli*, *P. aeruginosa*, and *K. pneumoniae* exhibited notable early growth in untreated cultures, whereas treated samples showed varied initial growth responses. For *E. coli*, an initial growth suppression was observed in hydrogel 1 : 1, while hydrogels at the 1 : 0 and 1 : 0.2 ratios showed some inhibition, though to a lesser extent. Gel 0.4 : 1, however, allowed substantial early growth, indicating that this formulation was less effective. In *P. aeruginosa* treated samples, hydrogels 1 : 0 and 1 : 0.2 were more effective at reducing early growth, while gels 1 : 1 and 0.4 : 1 allowed higher initial growth, suggesting that a higher Fmoc-Leu content may reduce early efficacy against this strain. For the *K. pneumoniae* strain, hydrogel 1 : 0 demonstrated the strongest initial inhibition, while gels 1 : 0.2 and 1 : 1 showed moderate suppression, and gel 0.4 : 1 had minimal effect, with OD_600_ values approaching those of the untreated controls. The results suggest that Gram-negative cells are more susceptible in the stationary phase than in the growth phase, as antimicrobial activity is observed during the stationary phase.

The initial growth rates indicate that formulations with higher Fmoc-Phe content, particularly in hydrogels 1 : 0 and 1 : 0.2, are more effective at immediately suppressing bacterial proliferation, especially against Gram-negative bacteria, where early growth inhibition can be critical for overall efficacy. The data suggest that Fmoc-Phe is likely the more effective component in inhibiting bacterial growth, as the hydrogels generally showed strong inhibitory effects across several bacterial strains, particularly against *K. pneumoniae* and *P. aeruginosa*. In contrast, formulations with a higher proportion of Fmoc-Leu (such as 0.4 : 1 gel) tend to be less effective, particularly against Gram-negative bacteria like *E. coli* and *K. pneumoniae*. Among the tested gel formulations, gels 1 : 0 and 1 : 0.2 appear to be the most effective in terms of broad-spectrum antimicrobial activity. Gel 1 : 0 consistently performed well against Gram-positive and Gram-negative bacteria, except for limited long-term inhibition against *E. faecalis*. Gel 1 : 0.2 also demonstrated strong inhibitory effects, particularly against *S. aureus*, *B. subtilis*, and *P. aeruginosa*, maintaining consistently low OD values.

These findings are consistent with previously proposed mechanisms for co-assembled Fmoc-Phe : Fmoc-Leu hydrogels. Irwansyah *et al.*^49^ reported that the antibacterial activity of such materials arises from contact-mediated disruption of the bacterial cell wall and membrane, driven primarily by hydrophobic interactions between exposed Fmoc/phenyl moieties and the bacterial envelope. The enhanced efficacy of Fmoc-Phe-rich formulations observed here supports this interpretation, suggesting that increased hydrophobic surface exposure contributes to more efficient perturbation of the bacterial membrane.^66,67^ Moreover, the reduced efficacy of Leu-enriched hydrogels aligns with the proposed role of Fmoc-Leu in a secondary, time-dependent interaction process involving its insertion or release upon bacterial contact. The selectivity observed toward Gram-positive strains in both studies further supports a mechanism in which leucine-rich motifs interact preferentially with the *S. aureus* cell wall.^49^ Collectively, these results reinforce the view that the antibacterial function of Fmoc-Phe : Fmoc-Leu hydrogels is primarily governed by hydrophobic contact-driven mechanisms, modulated by the relative composition of the two amino acid components.

## Conclusion

This work establishes a tuneable platform of dual-component Fmoc-Phe : Fmoc-Leu hydrogels that couple defined supramolecular organization with controllable antibacterial function. Spectroscopic and rheological analyses revealed that both components co-assemble into structurally consistent fibrillar networks dominated by π–π interactions of the Fmoc groups, while fluorescence lifetime data confirmed that Fmoc-Phe drives the core self-assembly process. Increasing Fmoc-Leu content softened the network (*G*′ decreasing from ∼600 Pa to ∼350 Pa) yet preserved reversible thermo-stiffening, with up to 30-fold modulus enhancement between 25 °C and 60 °C.

Quantitative antibacterial profiling uncovered a direct structure–activity relationship. Gram-positive strains (*S. aureus* and *B. subtilis*) were fully inhibited across all formulations (OD_600_ < 0.05), whereas *E. faecalis* remained suppressed for 48 h using 1 : 0.2 and 1 : 1 gels (OD_600_ < 0.2). Among Gram-negative species, *E. coli* and *P. aeruginosa* exhibited up to 55% growth reduction with 1 : 1 and 1 : 0.2 gels, and *K. pneumoniae* was most sensitive to Fmoc-Phe (1 : 0; OD_600_ ≈ 0.25). Hydrogels with elevated Fmoc-Leu content (0.4 : 1) lost activity across all strains (OD_600_ ≈ 0.8–1.0).

Collectively, the results show that Fmoc-Phe confers broad antimicrobial potency through hydrophobic contact-mediated disruption, while limited incorporation of Fmoc-Leu enhances persistence against Gram-positive species. The optimal formulations (1 : 0 and 1 : 0.2) achieve up to 100% inhibition of Gram-positive and 40–60% of Gram-negative pathogens. This work, therefore, defines how molecular composition governs both material mechanics and biological response, offering a rational framework for engineering peptide-based hydrogels with ratio-dependent antibacterial selectivity for biomedical and infection-control applications.

## Experimental details

### Materials

*N*-[(9*H*-Fluoren-9-ylmethoxy)carbonyl]-l-phenylalanine and *N*-[(9*H*-fluoren-9-ylmethoxy)carbonyl]-l-leucine were purchased from TCI chemicals and used without further purification. Fisher BioReagents™ phosphate buffer saline (PBS) tablets were used to prepare the PBS solution (pH = 7) for hydrogel preparation. PBS tablets were dissolved in either deionised water or D_2_O as appropriate.

### Gelation protocol

Transparent hydrogels were obtained by suspending either Fmoc-Phe and Fmoc-Leu or a combination of the two in 1 mL of PBS or D_2_O-PBS at the desired ratio, followed by several cycles of sonication and vortex until a fine suspension was obtained. The vials are heated at 80 °C in a block heater for >30 min, followed by shaking to resuspend the undissolved powder and an additional 30 min of heating to prevent particle decantation, resulting in particle-containing non-homogeneous hydrogels. The vials were left to rest undisturbed for >30 min at room temperature for gelation.

### Variable temperature NMR spectroscopy

The ^1^H NMR spectra of the gelator powders and corresponding hydrogels were obtained on a Bruker Advance III HD 500 MHz spectrometer. The samples were prepared using PBS dissolved in D_2_O as an NMR gelation solvent for *in situ* measurements. Chemical shifts (*δ*) are given in ppm, and the coupling constant (*J*) in Hz. All NMR spectra are solvent-suppressed to maximise sample detection. Variable temperature measurements were recorded from 30 °C to 70 °C with a 5 °C step and 5 min equilibration time.

### Infrared spectroscopy

The spectra of the gelator powders and the hydrogels were recorded on a Bruker Tensor 27 FT-IR spectrometer in attenuated total reflectance (ATR) mode and on a Nicolet iS50 FT-IR spectrometer in transmission mode. For measurements in ATR mode, hydrogels were dried in open air at room temperature, and spectra were recorded on the powder. For measurements in transmission mode, hydrogels were prepared *in situ* in the sample holder between 3 mm thick CaF_2_ windows. Gelation occurred in the holder, placed in a humid chamber. Spectral width: 400–4000 cm^−1^, number of scans: 64, resolution: 4 cm^−1^ for both techniques.

### Rheology measurements

Rheological measurements were performed on an Anton Paar MCR 302 modular compact rheometer with upper geometry cylinder ST10-4V-8.8/97.5. Hydrogels (1.0 mL) containing either Fmoc-Phe and Fmoc-Leu or a combination of the two were prepared in glass vials (Fisherbrand Type III soda lime glass, 14 mm inner diameter) with the geometry *in situ*. Frequency sweep measurements were performed within the linear viscoelastic region (0.00464%) obtained by amplitude sweep measurements. Temperature-controlled frequency sweep measurements were recorded from 25 °C to 60 °C at 5 °C intervals. Continuous step strain experiments were performed at 0.001% (low strain) and 1% (high strain), with a 15 minute recovery time between each run. All measurements were obtained in triplicate.

### UV-vis spectroscopy

The UV-visible spectra were obtained on a PerkinElmer Lambda 850 UV/vis spectrometer. The UV absorption profiles of the gelators were recorded at a concentration of 0.05 mg mL^−1^ in PBS solvent in a 1 mm path length quartz cuvette at room temperature. Spectral range: 250–500 nm, step: 1 nm, integration time: 0.2 s, slit width: 2 nm.

### Fluorescence spectroscopy

The steady-state fluorescence spectra were recorded on a Cary Eclipse fluorescence spectrometer on gelators and hydrogels in PBS solvent. The excitation wavelength was 289 nm in all measurements. Spectral range: 300–500 nm, excitation slit: 2.5 nm, emission slit: 5 nm, integration time: 0.4 s. The spectra were recorded in a 1 cm path length quartz cuvette at room temperature. Gels were prepared *in situ* within the cuvettes. Time-correlated single photon counting (TCSPC) measurements were performed using a UV-LED emitting at 290 nm (repetition rate set to 10 MHz). The polarisation on the excitation side was controlled by a polarising cube and a wire grid polariser at the analyser. Due to concentrated hydrogels, the excitation beam was focused on the corner of the cuvette to minimise the interaction with the molecules. The data points were collected within a 100 ns time window with a total collection time of 180 s.

### Transmission electron microscopy

Transmission electron microscope images were recorded on a JEOL JEM-1400HC microscope. Gel samples were placed onto the grid and allowed to dry in open air at room temperature overnight.

### Antibacterial activity

The antibacterial activity of the hydrogels was studied using *B. subtilis* VTT E-83177 (ATCC 21394), *E. coli* DSM613 (ATCC 11303), *S. aureus* (ATCC 25923), *K. pneumoniae* DSM681, E. faecalis NJ-3 (DSMZ12956), and *P. aeruginosa* PA14 (DSMZ19882). The bacteria were stored frozen with 10% glycerol at −80 °C. For the experiments, the bacteria were grown in L-media, except for *E. faecalis*, which was grown in trypticase soy yeast (TSB) extract medium (30 g TSB and 3.0 g yeast extract in one litre of water). The optical density (OD_600_) of overnight-grown bacteria was adjusted to 0.2 and diluted by 1 : 10 in the respective growth medium. 100 µL of this mixture was applied to the wells of two 96-well plates containing 100 µL of the different gel formulations (ratios 1 : 0, 1 : 0.2, 1 : 1 and 0.4 : 1). The hydrogels were replaced by PBS in bacterial growth controls, while the negative controls contained only sterile growth medium. The gel controls also contained a sterile growth medium. The plates were maintained at 37 °C under continuous shaking (orbital, 250 rpm). The optical density of each culture (OD_600_) was measured every 30 minutes over 48 hours using a Tecan Infinite 220 PRO M Nano plate reader.

All combinations were conducted in quadruplicate. For data analysis, three replicates with the closest OD_600_ values were selected from each set of four to reduce potential variability and ensure consistency in the measured antibacterial activity of each gel formulation. This selection method was applied uniformly across all experiments to maintain accuracy in data representation.

## Author contributions

R. C.: conceptualisation, formal analysis, investigation, validation, visualisation, writing – original draft, review & editing. H. R., E. D. S., T. K., L. J. W.: investigation, validation, writing – review & editing. L.-R. S.: resources, validation, writing – review & editing. J. R. H.: resources, validation, funding acquisition, writing – review & editing. M. P.: resources, funding acquisition, writing – review & editing. M. N.: supervision, validation, writing – review & editing.

## Conflicts of interest

The authors declare no conflicts of interest.

## Supplementary Material

RA-016-D5RA08809G-s001

## Data Availability

The original files are available from the corresponding author upon request. The data supporting this article have been included as part of the supplementary information (SI). Supplementary information: gelation screening, NMR/VT-NMR spectra, UV-vis spectra, fluorescence spectra, infrared spectra, rheology studies and TEM images. See DOI: https://doi.org/10.1039/d5ra08809g.
