# Supplementary material for: Fmoc-Phe : Fmoc-Leu supramolecular hydrogels with adaptive antibacterial activity
Source: RSC Adv. 2026 Mar 12;16(15):13801–11. doi: 10.1039/d5ra08809g (PMC12980527; doi:10.1039/d5ra08809g)
Supplement: RA-016-D5RA08809G-s001 [file RA-016-D5RA08809G-s001.pdf]

Supplementary Information (SI) for RSC Advances.
This journal is © The Royal Society of Chemistry 2026

Supporting Information

Fmoc-Phe:Fmoc-Leu Supramolecular Hydrogels with Adaptive Antibacterial Activity

Romain Chevigny,[a] Henna Rahkola,[a] Efstratios D. Sitsanidis,[a] Tatu Kumpulainen,[a] Lisa J. White,[b] Lotta-Riina Sundberg,[c] Jennifer R. Hiscock,[b] Mika Pettersson[a] and Maija Nissinen*[a]

[a] *Department of Chemistry, Nanoscience Center, University of Jyväskylä, FI-40014 Jyväskylä, Finland.*

[b] *School of Natural Sciences, University of Kent, Canterbury, Kent, CT2 7NH, UK.*

[c] *Department of Biological and Environmental Science, Nanoscience Center, University of Jyväskylä, FI-40014 Jyväskylä, Finland.*

Table of Contents

1. Gelation concentration screening 2
2. NMR spectroscopy characterisation 3
2.1. ^{1}H NMR spectrum of Fmoc-Phe 3
2.2. ^{1}H NMR spectrum of Fmoc-Leu 4
2.3. Variable temperature NMR 5
3. UV-vis measurements 6
4. Fluorescence spectroscopy 7
5. ATR and Transmission IR measurements 8
6. Rheology studies 10
7. Transmission electron microscopy 13

1. Gelation concentration screening

Table S1. Gelation concentration screening for dual gels depending on the ratio Fmoc-Phe:Fmoc-Leu and the required mass of each component. Phase transition temperature (°C) of the dual gels. Gels were formed in PBS or D_2O/PBS with a final volume of 1 mL.

Ratio (Fmoc-Phe : Fmoc-Leu)	Mass Fmoc-Phe (mg)	Mass Fmoc-Leu (mg)	Outcome	$T_{gel\text{-}sol}$ (°C)
1 : 0	2	0	SSG, transparent	60
1 : 0.2	1.66	0.33	SSG, transparent	55-60
1 : 1	1	1	SSG, transparent	50-55
0.4 : 1	0.57	1.42	SSG, transparent	45-50
0 : 1	0	2	Precipitate	/[a]

[a] Not measured.

2. NMR spectroscopy characterisation

2.1. ^{1}H NMR spectrum of Fmoc-Phe

^{1}H NMR (500 MHz, D_2O) δ 7.98 (t, J = 6.5 Hz, 2H), 7.70 (dd, J = 11.4, 7.5 Hz, 2H), 7.56 (t, J = 7.6 Hz, 2H), 7.48 (q, J = 7.0 Hz, 2H), 7.33 (dd, J = 14.5, 7.1 Hz, 2H), 7.17 (d, J = 7.3 Hz, 1H), 7.01 (s, 1H), 4.40 (dd, J = 10.8, 5.7 Hz, 1H), 4.29 (s, 1H), 4.20 (s, 1H), 3.11 (dd, J = 14.0, 4.6 Hz, 1H), 2.85 (dd, J = 13.8, 8.7 Hz, 1H).

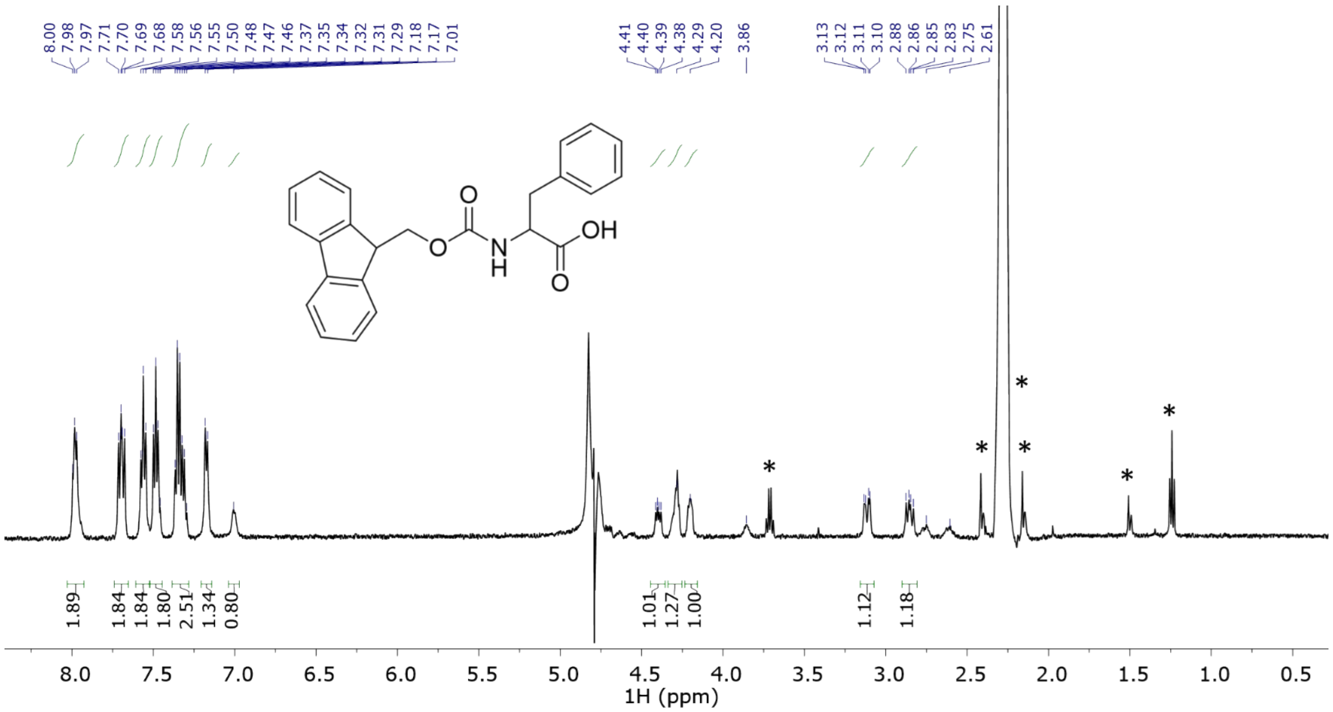

Figure S1. ^{1}H NMR (500 MHz) spectrum of Fmoc-Phe. The solvent signal (D_2O) at 4.79 ppm was suppressed to maximise the sample signals. Solvent impurities and ^{13}C satellites are marked with asterisks.

2.2. ^{1}H NMR spectrum of Fmoc-Leu

^{1}H NMR (500 MHz, D_2O) δ 7.98 (dd, *J* = 7.6, 4.8 Hz, 2H), 7.80 (d, *J* = 7.5 Hz, 1H), 7.75 (d, *J* = 7.4 Hz, 1H), 7.57 (td, *J* = 7.4, 4.4 Hz, 2H), 7.49 (q, *J* = 8.4, 7.8 Hz, 2H), 4.55 (dd, *J* = 11.0, 5.1 Hz, 1H), 4.39 (t, *J* = 5.4 Hz, 1H), 3.88 (dd, *J* = 10.5, 4.2 Hz, 1H), 1.49 – 1.38 (m, 3H), 0.87 (dd, *J* = 26.9, 6.4 Hz, 6H).

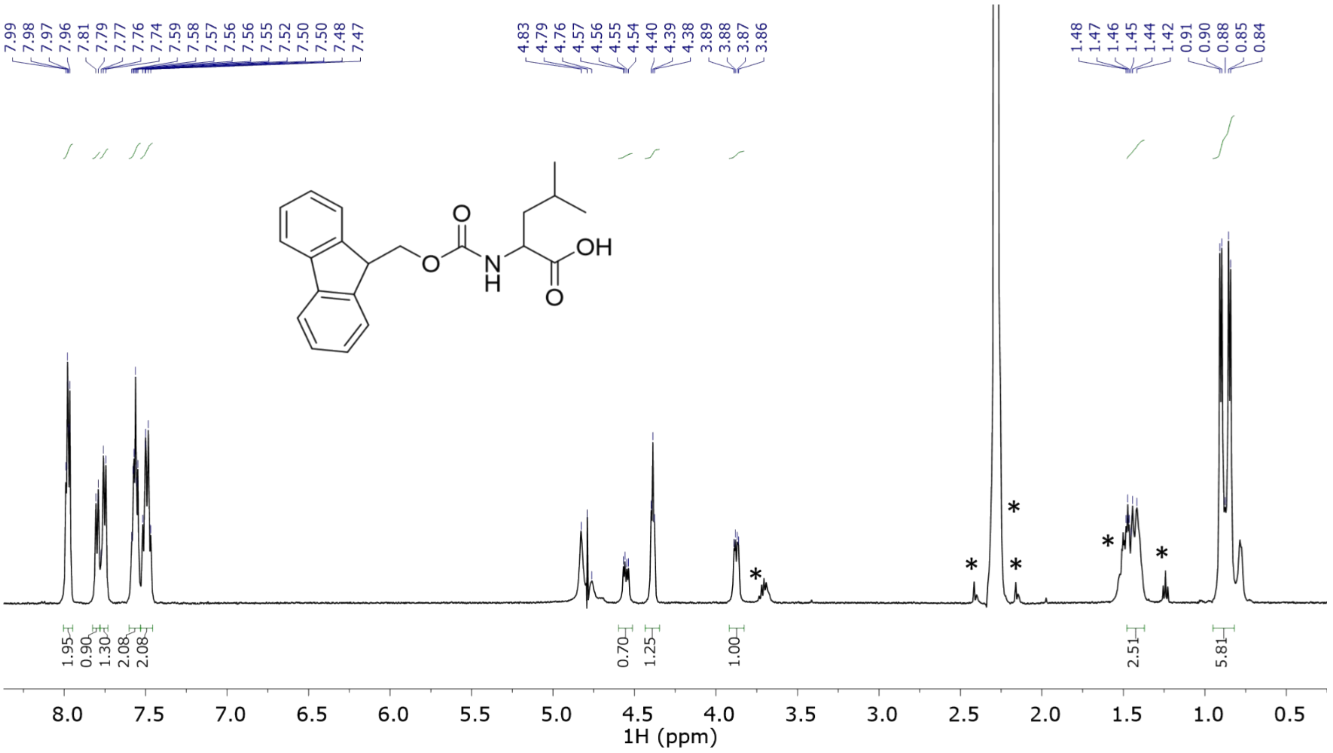

Figure S2. ^{1}H NMR (500 MHz) spectrum of Fmoc-Leu. The solvent signal (D_2O) at 4.79 ppm was suppressed to maximise the sample signal. Solvent impurities and ^{13}C satellites are marked with asterisks.

2.3. Variable temperature NMR

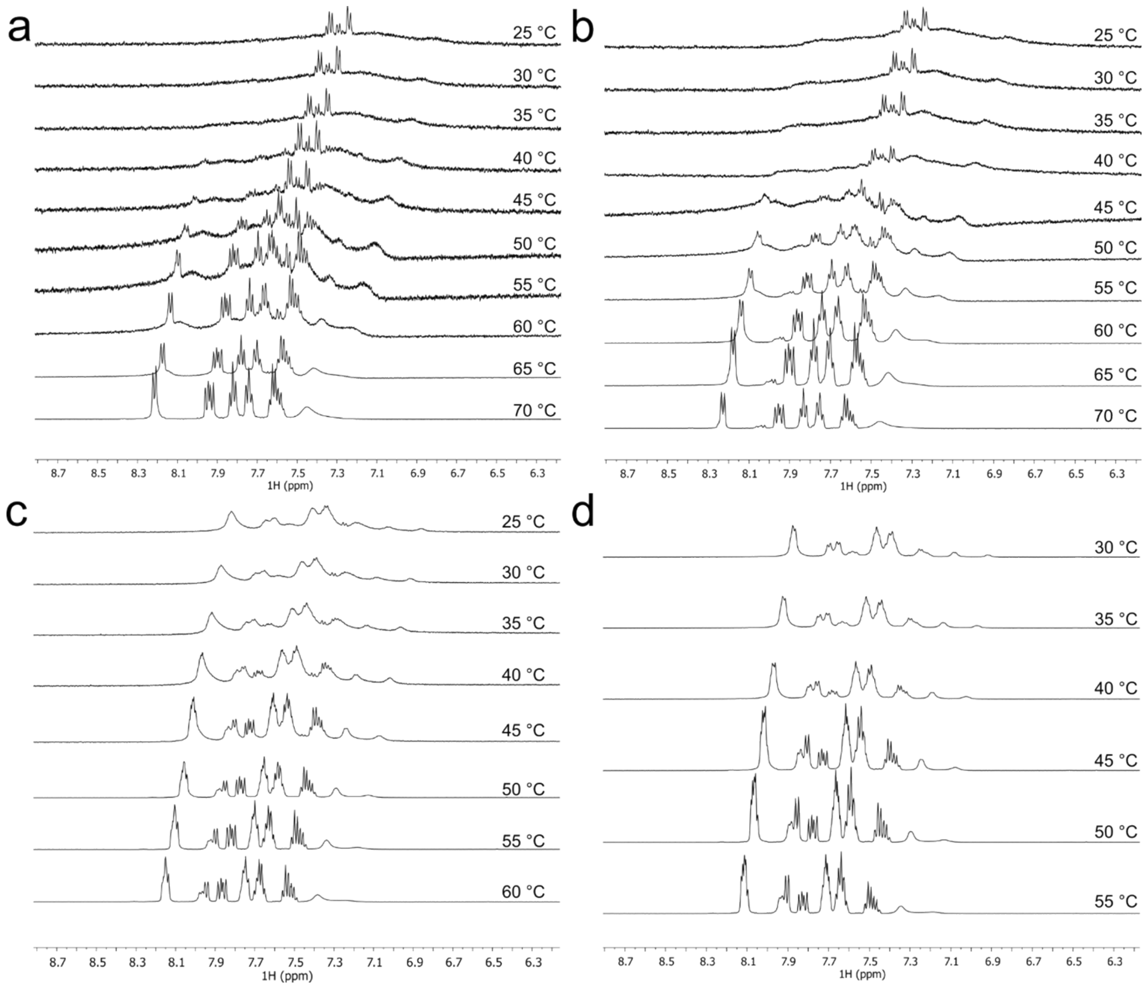

Figure S3. Variable temperature ^{1}H NMR spectra of Fmoc-Phe:Fmoc-Leu gels in D_2O/PBS at ratios a) 1:0, b) 1:0.2, c) 1:1 and d) 0.4:1. The spectra are magnified in the aromatic region (6.3 – 8.7 ppm). Spectra were recorded at 5 °C intervals with a 5 min equilibration time. The temperature increase was stopped at the point at which the peak sharpened.

3. UV-vis measurements

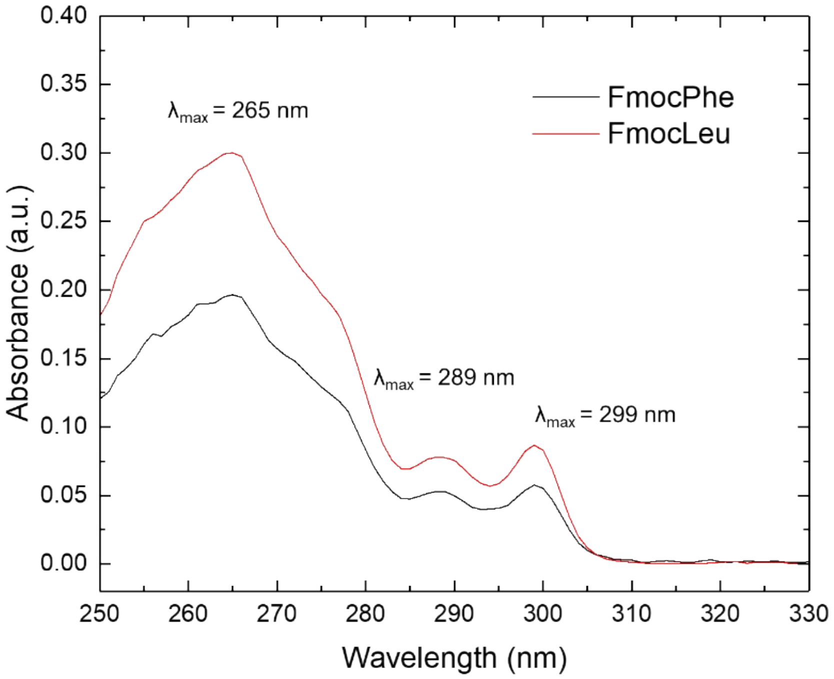

Figure S4. UV-vis spectra of Fmoc-Phe and Fmoc-Leu at 0.05 mg/mL in PBS solution. Spectra were recorded at room temperature.

4. Fluorescence spectroscopy

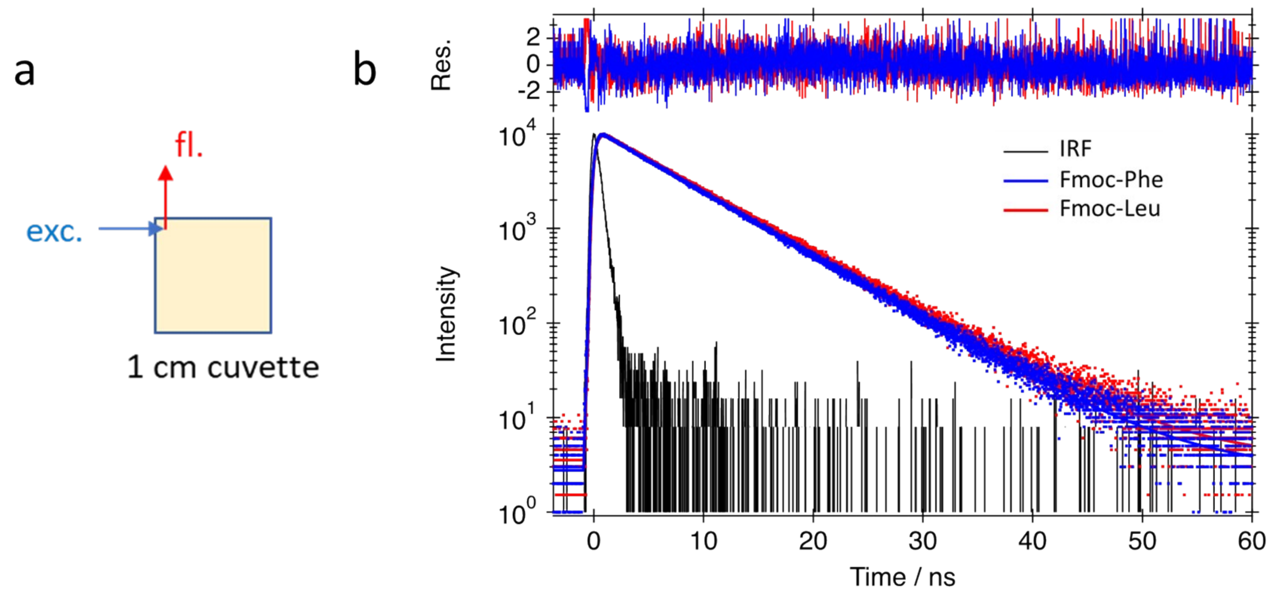

Figure S5. a) A schematic depiction of the collection geometry of the TCSPC setup. Because of the high absorbance of the hydrogels, the excitation beam is directed to the corner of the cuvette to minimise self-absorption. b) Fluorescence decay curves of the monomers in solution and the instrument response function (IRF) measured directly from the scattered excitation light in pure water.

Table S2. Two-exponential fit parameters of the fluorescence decays of Fmoc-Phe and Fmoc-Leu in PBS solution.

Compound	a_1 (%)	t_1 (ps)	a_2 (%)	t_2 (ns)	chi^2
**Fmoc-Phe**	25	600 +/- 140	75	6.5 +/- 0.1	1.107
**Fmoc-Leu**	28	490 +/- 150	72	6.7 +/- 0.1	1.109

Table S3. Three-exponential fit parameters of the fluorescence decays of the dual-component gels in PBS with different Fmoc-Phe:Fmoc-Leu ratios.

Ratio	a_1 (%)	t_1 (ps)	a_2 (%)	t_2 (ns)	a_3 (%)	t_3 (ns)	chi^2
**1:0**	74	430 +/- 50	21	4.8 +/- 0.2	5	12.6 +/- 0.7	1.263
**1:0.2**	67	510 +/- 60	29	5.6 +/- 0.2	4	15 +/- 1	1.138
**1:1**	61	340 +/- 60	36	6.4 +/- 0.2	4	19.7 +/- 1.2	1.106
**0.4:1**	55	290 +/-60	41	6.5 +/- 0.2	4	20.8 +/- 1.1	1.107

5. ATR and Transmission IR measurements

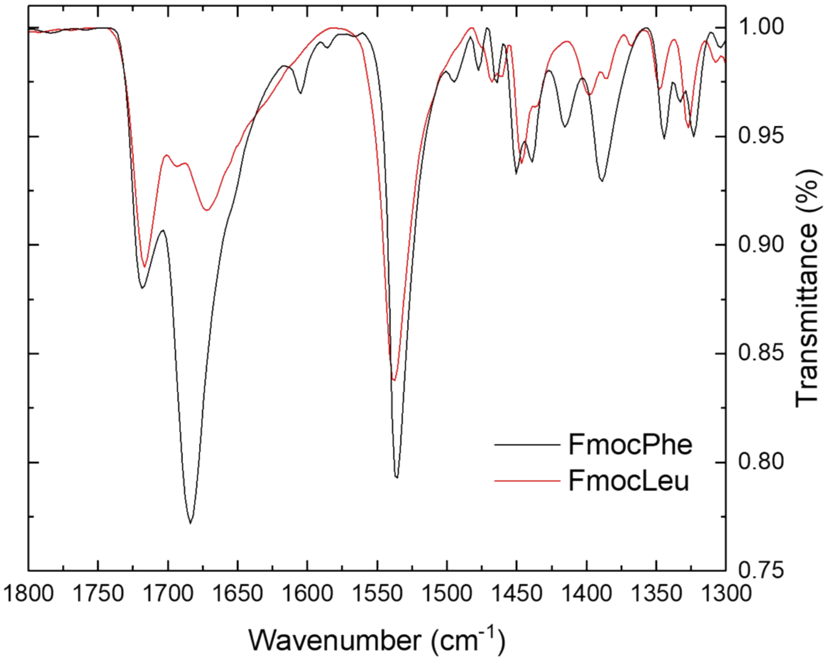

Figure S6. ATR spectra of Fmoc-Phe and Fmoc-Leu gelators.

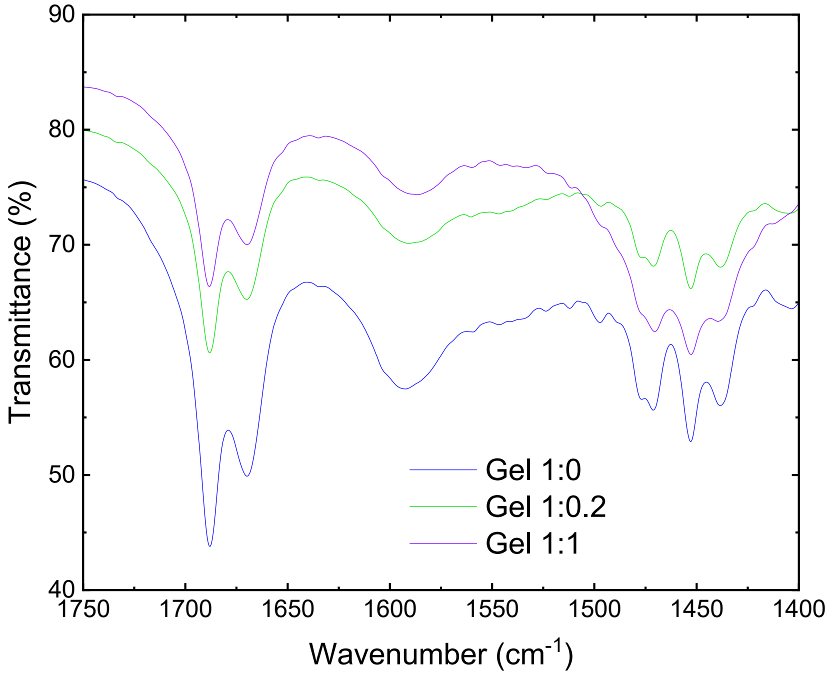

Figure S7. Transmission IR spectra of Fmoc-Phe/Fmoc-Leu dual hydrogels with different ratios Fmoc-Phe:Fmoc-Leu in the range 1720–1640 cm^{-1}. Gels were made in vials, mechanically broken, and let to reform for 15 min between the CaF_2 windows before the measurement.

a b c d

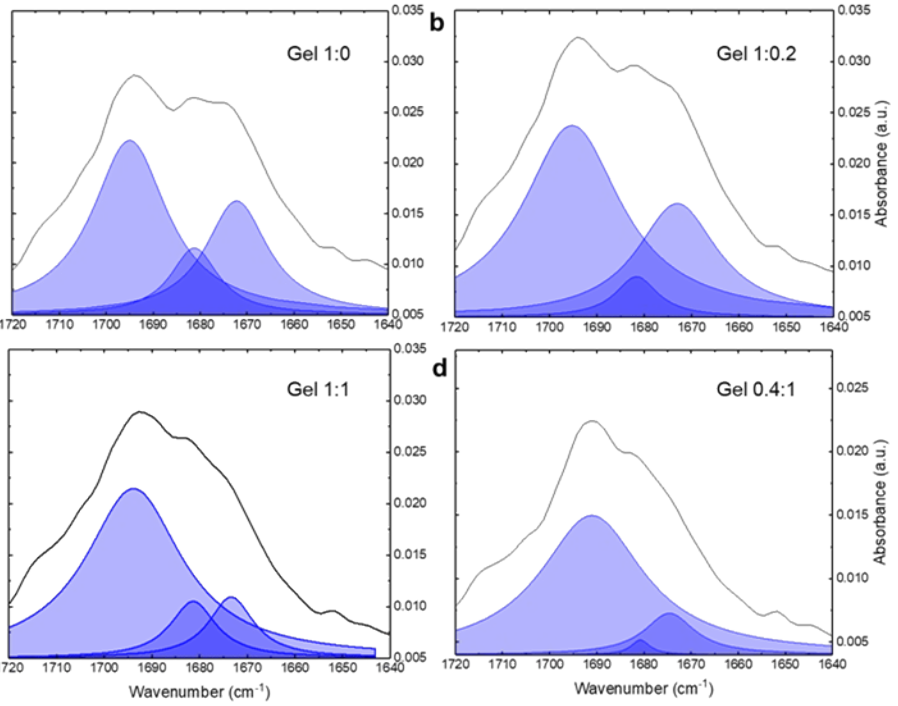

e

Gel 1:0		Gel 1:0.2	
Peak position (cm-1)	Composition (%)	Peak position (cm-1)	Composition (%)
1694	55	1694	62
1681	15	1681	7
1672	30	1673	31

Gel 1:1		Gel 0.4:1	
Peak position (cm-1)	Composition (%)	Peak position (cm-1)	Composition (%)
1694	73	1694	84
1681	13	1681	2
1673	14	1673	14

f

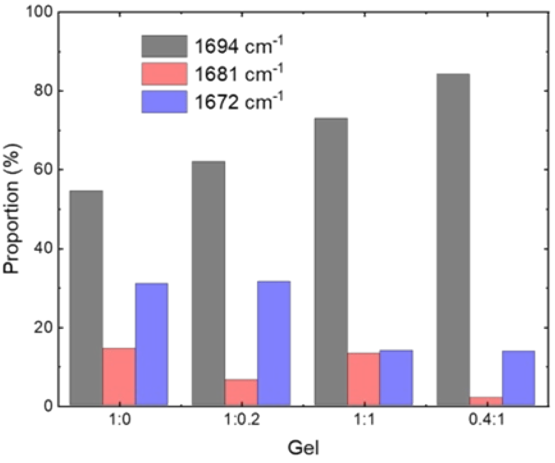

Figure S8. a-d) ATR-FTIR spectra of Fmoc-Phe/Fmoc-Leu dual hydrogels with different ratios Fmoc-Phe:Fmoc-Leu in the range 1720–1640 cm^{-1}. Blue curves are the fitted bands with a Lorentzian shape upon deconvolution. e) Summary of the three main peaks composing the convoluted band for each gel system, and f) the bar graph representation

6. Rheology studies

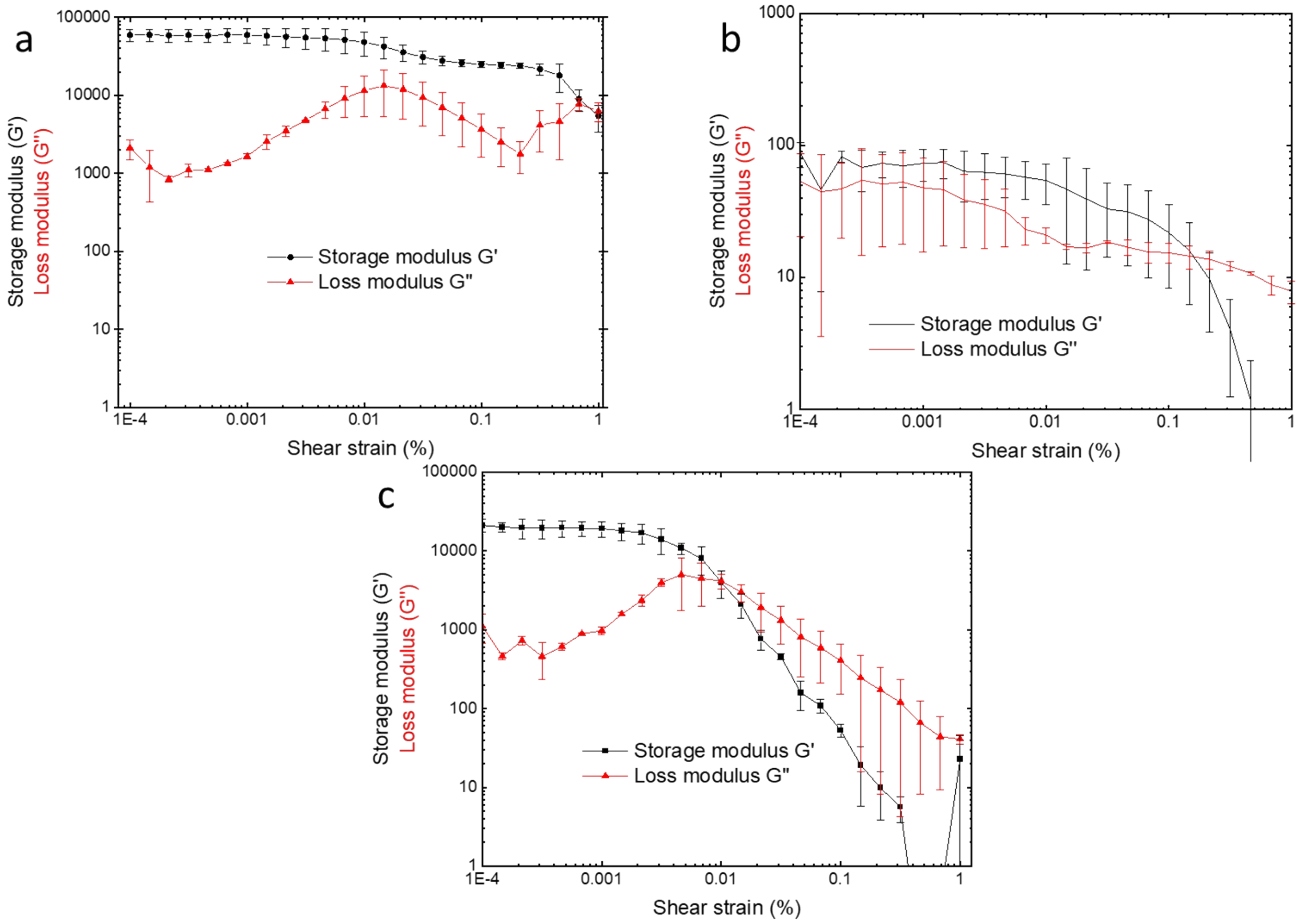

Figure S9. Amplitude sweep measurement of gel ratio a) 1:0, b) 1:1 and c) 0.4:1 at room temperature. The linear viscoelastic region is 0.00464% shear strain for all.

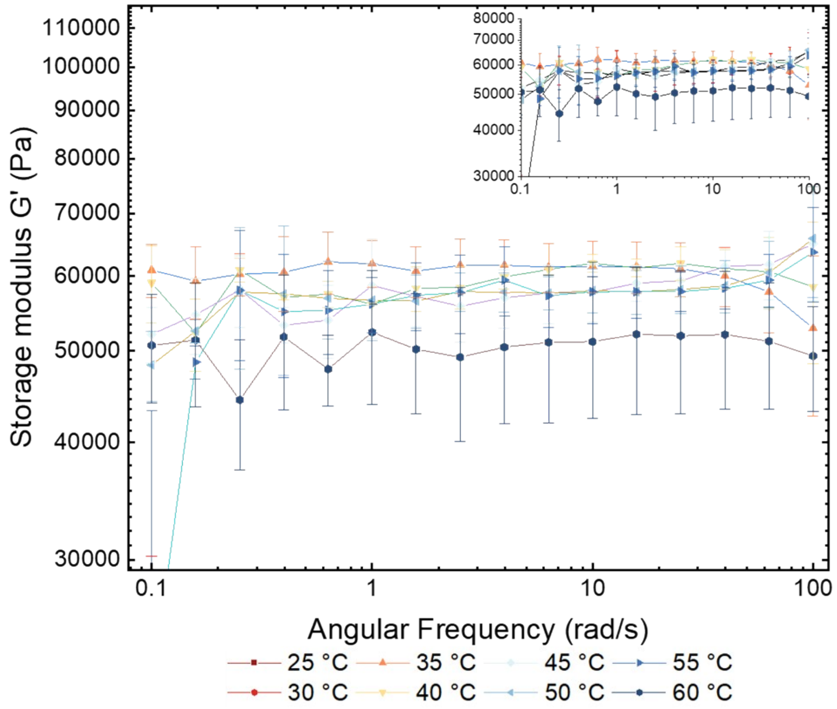

Figure S10. Temperature-dependent frequency sweep at 0.00464% shear strain of gel ratio 1:0 from 25 °C to 60 °C. Gel samples were left for ~15 min to reach equilibrium before recording.

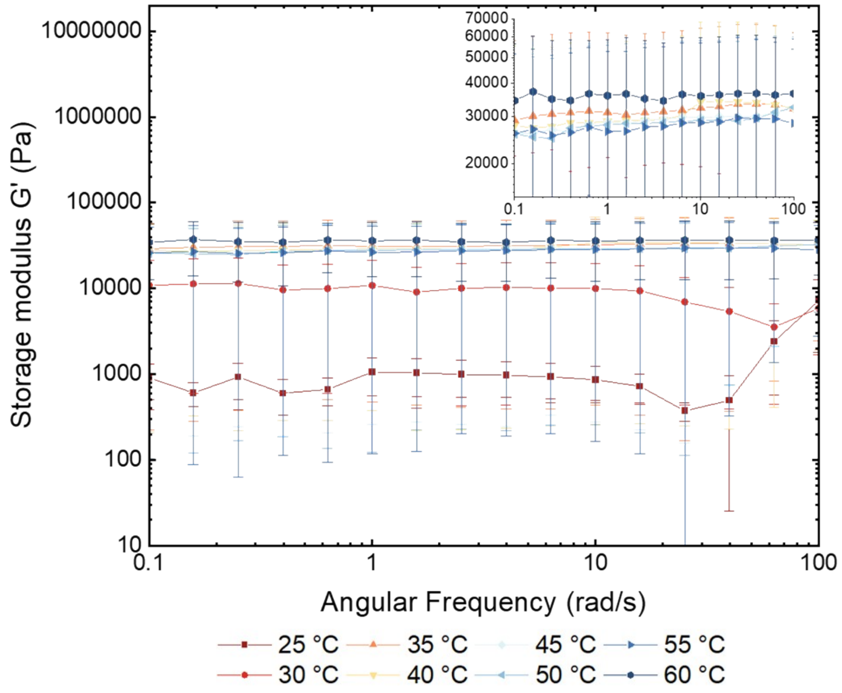

Figure S11. Temperature-dependent frequency sweep at 0.00464% shear strain of gel ratio 1:1 from 25 °C to 60 °C. Gel samples were left for ~15 min to reach equilibrium before recording. The lower error bars in the inset are not fully visible.

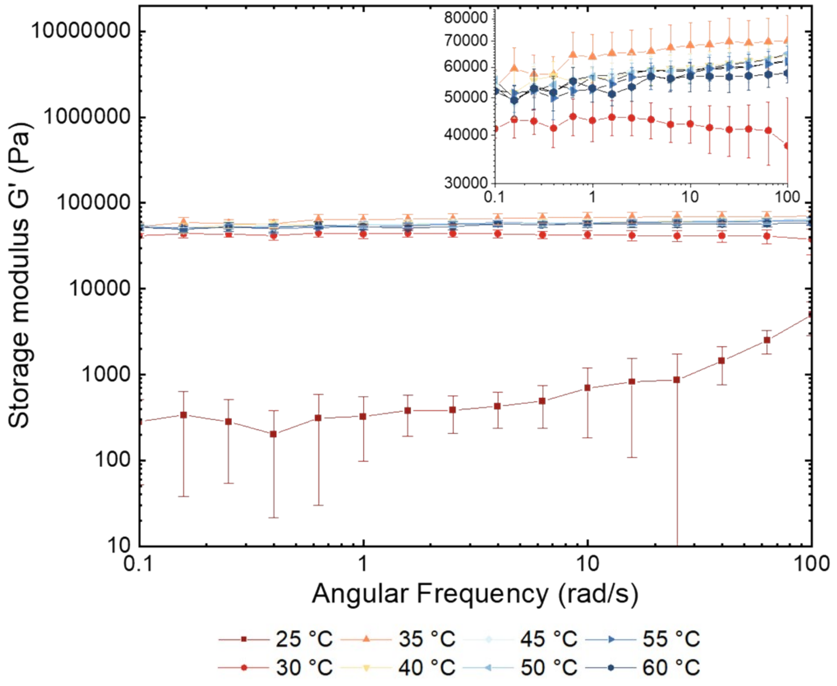

Figure S12. Temperature dependant frequency sweep at 0.00464% shear strain of gel ratio 0.4:1 from 25 °C to 60 °C. Gel samples were left ~15 min to reach equilibrium before recording.

7. Transmission electron microscopy

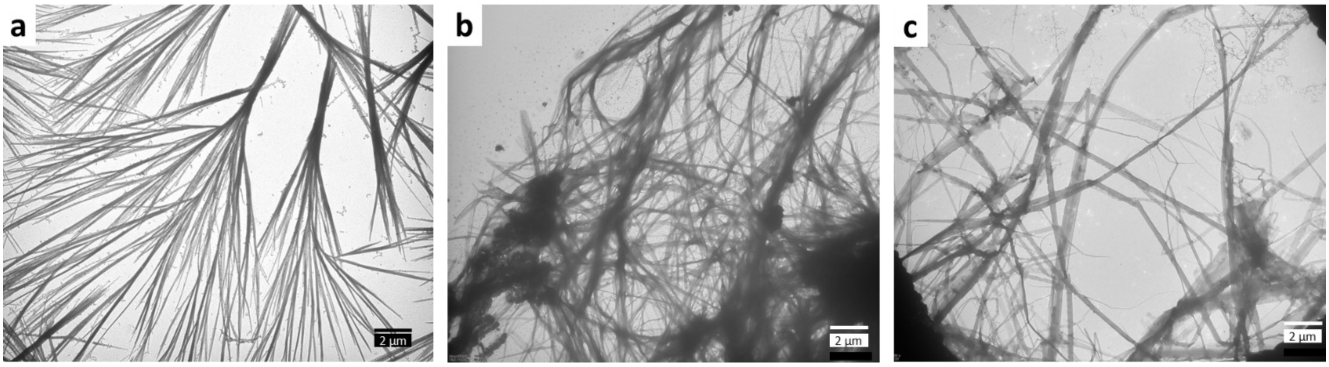

Figure S13. TEM images of gel ratio 1:0 (a), 1:1 (b), and 0.4:1 (c). Samples were freeze-dried before imaging.
